# FoxH1 Represses the Promoter Activity of *cyp19a1a* in the Ricefield Eel (*Monopterus albus*)

**DOI:** 10.3390/ijms241813712

**Published:** 2023-09-05

**Authors:** Zhi He, Qiqi Chen, Jinxin Xiong, Mingqiang Chen, Kuo Gao, Bolin Lai, Wenxiang Ding, Junjie Huang, Li Zheng, Yong Pu, Ziting Tang, Mingwang Zhang, Deying Yang, Taiming Yan

**Affiliations:** College of Animal Science and Technology, Sichuan Agricultural University, Chengdu 611130, China; zhihe@sicau.edu.cn (Z.H.); 2021202075@stu.sicau.edu.cn (Q.C.);

**Keywords:** FoxH1, *cyp19a1a* promoter, *Monopterus albus*, immunocolocalization, gonadotropins

## Abstract

Forkhead box H1 (FoxH1) is a sexually dimorphic gene in *Oreochromis niloticus*, *Oplegnathus fasciatus*, and *Acanthopagrus latus*, indicating that it is essential for gonadal development. In the present study, the molecular characteristics and potential function of FoxH1 and the activation of the *cyp19a1a* promoter in vitro were evaluated in *Monopterus albus*. The levels of *foxh1* in the ovaries were three times higher than those in the testes and were regulated by gonadotropins (Follicle-Stimulating Hormone and Human Chorionic Gonadotropin). FoxH1 colocalized with Cyp19a1a in the oocytes and granulosa cells of middle and late vitellogenic follicles. In addition, three FoxH1 binding sites were identified in the proximal promoter of *cyp19a1a*, namely, FH1 (−871/−860), FH2 (−535/−524), and FH3 (−218/−207). FoxH1 overexpression significantly attenuated the activity of the *cyp19a1a* promoter in CHO cells, and FH1/2 mutation increased promoter activity. Taken together, these results suggest that FoxH1 may act as an important regulator in the ovarian development of *M. albus* by repressing *cyp19a1a* promoter activity, which provides a foundation for the study of FoxH1 function in bony fish reproductive processes.

## 1. Introduction

Ovarian follicular cell proliferation and differentiation are critical processes in follicle development that are regulated by multiple endocrine, paracrine, and autocrine factors. These factors simultaneously enhance the crosstalk between oocytes and follicular cells. In teleost ovaries, interstitial cells, granulosa cells (GCs), and thecal cells (TCs) mediate steroidogenesis due to the abundance of intracellular steroidogenic enzymes, such as CYP11, CYP17, and CYP19 [1,2]. Previous studies have shown that fox transcription factors regulate the promoter activity of *cyp19a1* through a conserved N-terminal Forkhead domain that binds to DNA [3,4]. In teleosts, FoxL2 [5,6,7], FoxO1 [8], and FoxO3 [9] bind to the *cyp19a1a* promoter via the Forkhead domain and significantly upregulate its transcriptional activity. In addition, the conserved potential binding sites of FoxO4, FoxF1/2, and FoxL1 in the *cyp19a1a* promoter have been predicted [10,11]. Some *fox* genes are sexually dimorphic, including *foxj2*, *foxj1a*, and *foxh1*, and even exhibit male-specific expression [12]. These results indicate that fox genes are widely involved in sex determination and folliculogenesis.

Forkhead box H1 (FoxH1) is a critical Smad2/3 cofactor [13,14] involved in regulating gene expression downstream of Activin/Nodal/TGF-β signals during early embryo development [15,16]. FoxH1 interacts with Smad2/3/4 via the C-terminal Smad interaction domain (SID) to form the Smad/FoxH1 complex, which binds to DNA via a conserved N-terminal Forkhead domain and regulates target gene transcription [17]. The presence of *FoxH1* mRNA in the unfertilized eggs of Xenopus [18], zebrafish [19], and mice [20] indicates that this gene is maternally expressed. In mouse ovaries, FoxH1 is expressed in oocytes and TCs, but FoxH1 is expressed specifically in TCs during ovarian follicle development, ovulation, and luteinization [21]. In *Oplegnathus fasciatus* [22], *Acanthopagrus latus* [23], and *Oreochromis niloticus* [12], foxh1 is a sexually dimorphic gene, and its expression level in the ovaries is higher than that in the testes. These findings reveal that *foxh1* might play essential roles during ovarian development.

The ricefield eel (*Monopterus albus*) is a protogynous hermaphrodite fish with few oocytes and low fecundity that is increasingly being used as a model vertebrate for the study of gonad development and sex change [24,25]. Our previous RNA-seq data showed that *foxh1* expression was highest in the ovaries, followed by the intersex gonads, and was very low in the testes [26]. In addition, *foxh1*^−/−^ XX tilapia oogenesis was arrested, and Cyp19a1a expression was markedly decreased. However, the mechanism by which FoxH1 regulates the expression of *cyp19a1a* in *M. albus* remains unclear. To explore the function of FoxH1 in follicle development and the transcriptional regulation of *cyp19a1a* in *M. albus*, the sequences and expression patterns of FoxH1 were determined, and ovarian tissue fragments were incubated with gonadotropin in vitro. Then, the localization of FoxH1 and Cyp19a1a in the ovaries was analyzed. Finally, the mechanism whereby FoxH1 transcriptionally regulates *cyp19a1a* was analyzed. We found that FoxH1 plays a critical role in follicular cell differentiation through possible effects on *cyp19a1a* transcription and estrogen synthesis in ricefield eel.

## 2. Results

### 2.1. Sequence Analysis of Ricefield Eel foxh1

**Figure 1:**
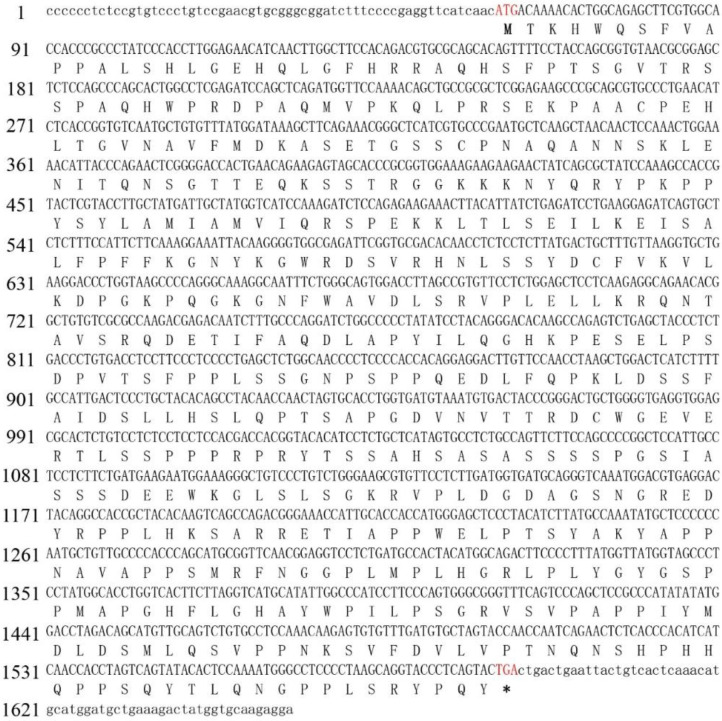
Nucleotide and amino acid sequences of the coding region of FoxH1 in the ricefield eel, *Monopterus albus*. The untranslated regions and translated regions are indicated by lowercase letters and uppercase letters, respectively. The initiation codon (ATG) and stop codon (TGA) are marked in red. Asterisks (*) indicate the translation stop codon.

**Figure 2:**
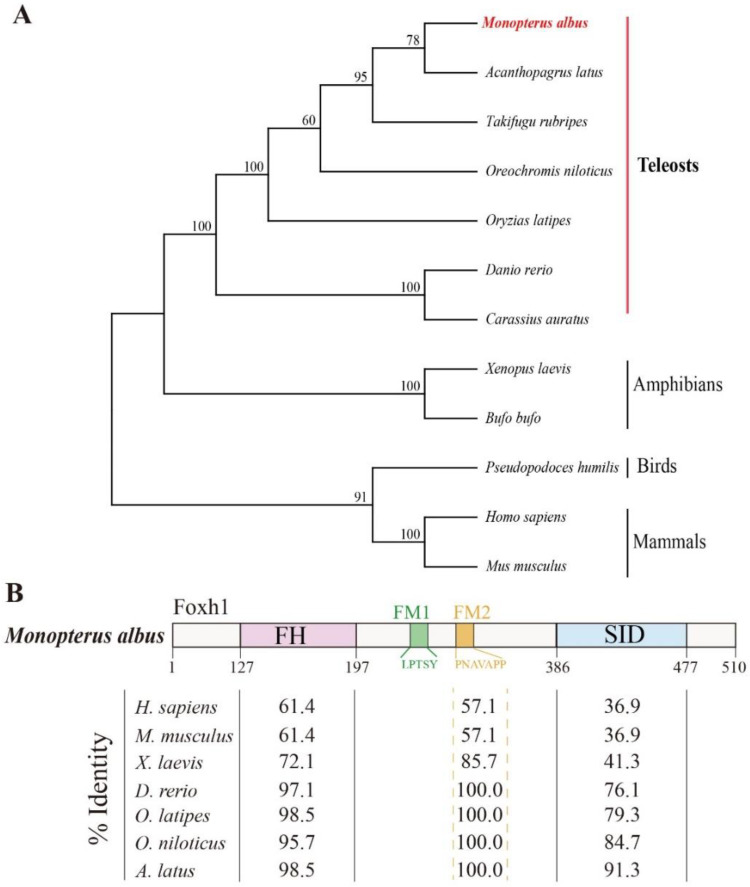
Phylogenetic analysis and domain characteristics of FoxH1 in the ricefield eel, *Monopterus albus*. (**A**) Neighbor-joining phylogenetic trees of FoxH1. The numbers at the nodes are bootstrap proportions. Other vertebrate FoxH1 protein sequences were downloaded from the NCBI database. (**B**) The characteristic FoxH1 domains are conserved in ricefield eel orthologs. The numbers represent the percent identities of the predicted protein sequences with other FoxH1 orthologs. FH: Forkhead domain; FM1/2: FoxH1 domain 1/2; SID: Smad interaction domain. (*Homo sapiens*, NP_003914.1; *Mus musculus*, NP_032015.1; *Xenopus laevis*, NP_001081820.1; *Danio rerio*, NP_571577.1; *Oryzias latipes*, NP_001153943.1; *Oreochromis niloticus*, XP_003443542.1; *Acanthopagrus latus*, XP_036934497.1).

The coding sequence of *foxh1* was 1533 base pairs (bp) in length and consisted of three exons (encoding a putative protein of 510 amino acids) (Figure 1). A phylogenetic tree was constructed based on the full protein sequences of FoxH1 orthologs. FoxH1 of the ricefield eel clustered with those of other teleost species and clustered closely with the sequence of *A. latus* (Figure 2A). Consistent with the FoxH1 orthologs of other vertebrates, FoxH1 contains three typical domains: a Forkhead domain, a FoxH1 domain (FM1/2), and an SID. In the ricefield eel, the FoxH1 domain was the most conserved, followed by the Forkhead domain and the SID. In mammals, amphibians, and teleosts, the FM1 domain was fairly conserved, but the FM2 domain was not (Figure 2B).

### 2.2. Expression Patterns of foxh1 in M. albus

**Figure 3:**
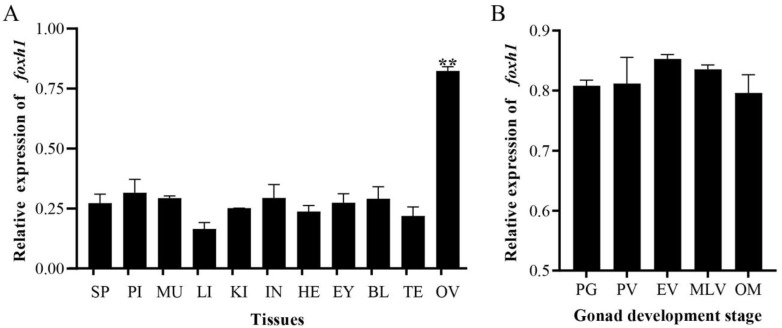
Expression levels of *foxh1* in different tissues and during ovarian development in *Monopterus albus*. (**A**) Relative mRNA levels of *foxh1* in tissues. (**B**) Relative *foxh1* mRNA levels during ovarian development. SP, spleen; PI, pituitary; MU, muscle; LI, liver; KI, kidney; IN, intestine; HE, heart; EY, eye; BL, blood; TE, testis; OV, ovary. The results are presented as the means ± SEMs (*n* = 4). ** p < 0.01.

*foxh1* mRNA was widely expressed in all examined tissues, including the brain, heart, liver, kidney, intestine, spleen, blood, ovary, and testis. The highest levels of *foxh1* transcripts were detected in the ovaries, where they were approximately three times higher than those in the testes (*p* < 0.01) (Figure 3A). There were no significant differences in *foxh1* expression levels during the five stages of ovarian development (*p* > 0.05) (Figure 3B). However, *foxh1* expression levels increased from the primary growth (PG) to early vitellogenic (EV) stages and decreased from the EV to the mature ovary (OM) stages. They were highest in the EV stage, followed by the mid-to-late vitellogenic (MLV) stage. These results suggest that *foxh1* is a sexually dimorphic gene that may play a crucial role in female sexual cycle maintenance and ovarian vitellogenesis.

### 2.3. Expression of foxh1 after FSH and hCG Incubation In Vitro

**Figure 4:**
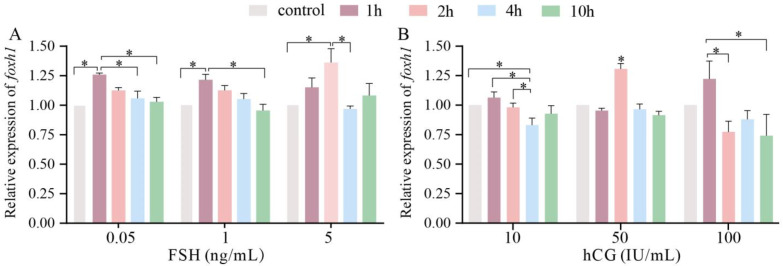
Regulation of *foxh1* expression in the ovary by FSH and hCG in vitro. (**A**) Expression levels of *foxh1* after FSH incubation. (**B**) Expression levels of *foxh1* after hCG incubation. The results are presented as the means ± SEMs (*n* = 5). FSH, follicle stimulating hormone; hCG, human chorionic gonadotropin; * *p* < 0.05.

Overall, FSH and hCG had different stimulatory effects on *foxh1* expression levels. In the 0.05 ng/L and 1 ng/L FSH groups (Figure 4A), the expression levels of *foxh1* had a similar pattern of change, in which *foxh1* levels were significantly elevated at 1 h (*p* < 0.05) and then slowly decreased to the control level. The highest concentration of FSH (5 ng/L) significantly increased the *foxh1* expression at 2 h (*p* < 0.05). Furthermore, in the 10 IU/mL hCG group, the *foxh1* expression levels decreased significantly at 4 h (*p* < 0.05) (Figure 4B). In the 50 IU/mL hCG group, the *foxh1* level increased significantly at 2 h compared to other time points (*p* < 0.05). In the 100 IU/mL hCG group, *foxh1* expression levels increased at 1 h but were not significant. However, *foxh1* expression levels at 2 h, 4 h, and 10 h were lower than those of the control.

### 2.4. Colocalization of FoxH1/Cyp19a1a in EV-Stage M. albus Ovaries

**Figure 5:**
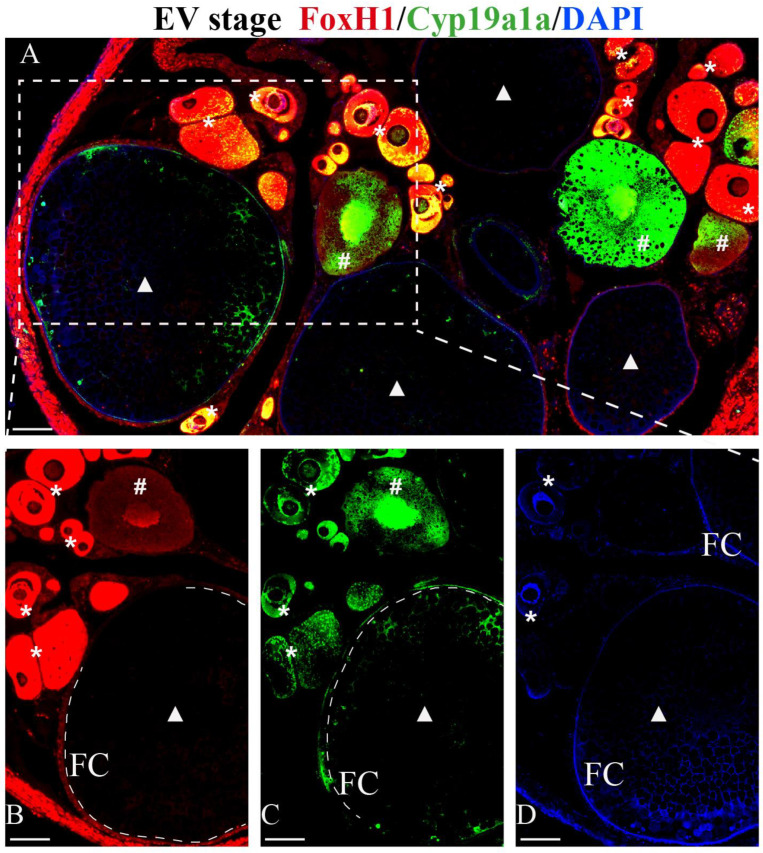
Colocalization of immunofluorescent FoxH1 (red) and Cyp19a1a (green) signals in EV Monopterus albus ovaries. (**A**) Colocalization of Foxh1 and Cyp19a1a immunostaining. (**B**) Foxh1 immunostaining. (**C**) Cyp19a1a immunostaining. (**D**) Nuclei are labeled with DAPI (blue). (**B**–**D**) are the dotted boxes in Figure 5A. Asterisk (*), primary growth oocytes (PGOs); pound (#), cortical alveoli stage oocytes (CAOs), triangle (Δ), early vitellogenic-stage oocytes (EVOs); FC, follicular cells; Nu, nuclei. The scale bar is 100 µm.

The subcellular colocalization of FoxH1 and Cyp19a1a in EV ovaries was determined by immunofluorescence (Figure 5). FoxH1 was mainly localized in the nuclei and cytoplasm of primary growth oocytes (PGOs) (Figure 5B and Figure S1). However, FoxH1 was present only in the follicular cells of EV follicles (Figure 5B). Cyp19a1a was localized in the nuclei and cytoplasm of PGOs, cortical alveoli stage oocytes (CAOs), and early vitellogenic-stage oocytes (EVOs) (Figure 5A,C and Figure S2). It was also localized in the follicular cell nuclei of EV follicles (Figure 5C), and the fluorescence signal of Cyp19a1a was stronger than that of FoxH1 in follicular cells. In general, FoxH1 and Cyp19a1a colocalized in the cytoplasm and nuclei of PGOs, the cytoplasm of EVOs, and the follicular cells of EV follicles (Figure 5A).

### 2.5. Colocalization of FoxH1 and Cyp19a1a in MLV-Stage M. albus Ovaries

**Figure 6:**
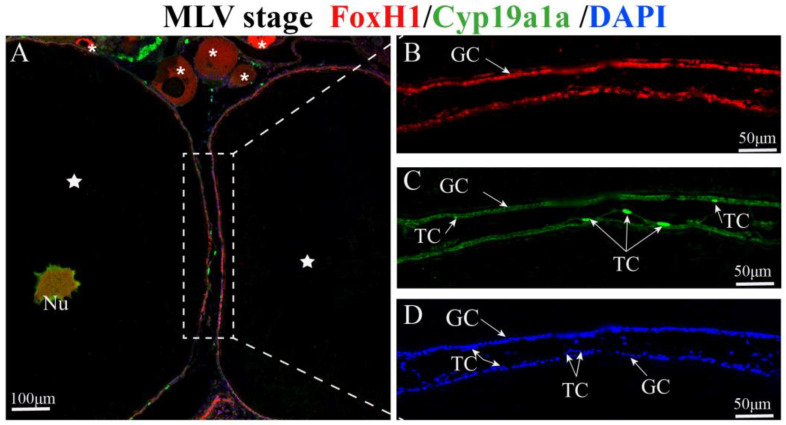
Immunofluorescent colocalization of Foxh1 (red) and Cyp19a1a (green) signals in MLV Monopterus albus ovaries. (**A**) Colocalization of FoxH1 and Cyp19a1a immunostaining. (**B**) FoxH1 immunostaining. (**C**) Cyp19a1a immunostaining. (**D**) Nuclei are labeled with DAPI (blue). (**B**–**D**) are the dotted boxes of Figure 6A enlarged. Asterisk (*), primary growth oocytes (PGOs); pentagram (☆), middle to late vitellogenic-stage oocytes (MLVOs); FC, follicular cells; GC, granulosa cells; TC, thecal cells; Nu, nuclei.

The subcellular colocalization of FoxH1 and Cyp19a1a in MLV ovaries was examined based on immunofluorescence (Figure 6). In MLV follicles, follicular cells proliferate and differentiate to form inner GCs and outer TCs (Figure 6D). FoxH1 was localized in the nuclei of oocytes and GCs. Cyp19a1a was localized in the nuclei of oocytes, GCs, and TCs (Figure 6A,C). FoxH1 and Cyp19a1a colocalized in the nuclei of oocytes and GCs, whereas no fluorescent signal was observed in the cytoplasm of oocytes (Figure 6A). Moreover, the specific signal of Cyp19a1a was stronger than that of FoxH1 in TCs (Figure 6C).

### 2.6. Activation of the cyp19a1a Promoter by Foxh1 via the Forkhead Binding Site In Vitro

**Figure 7:**
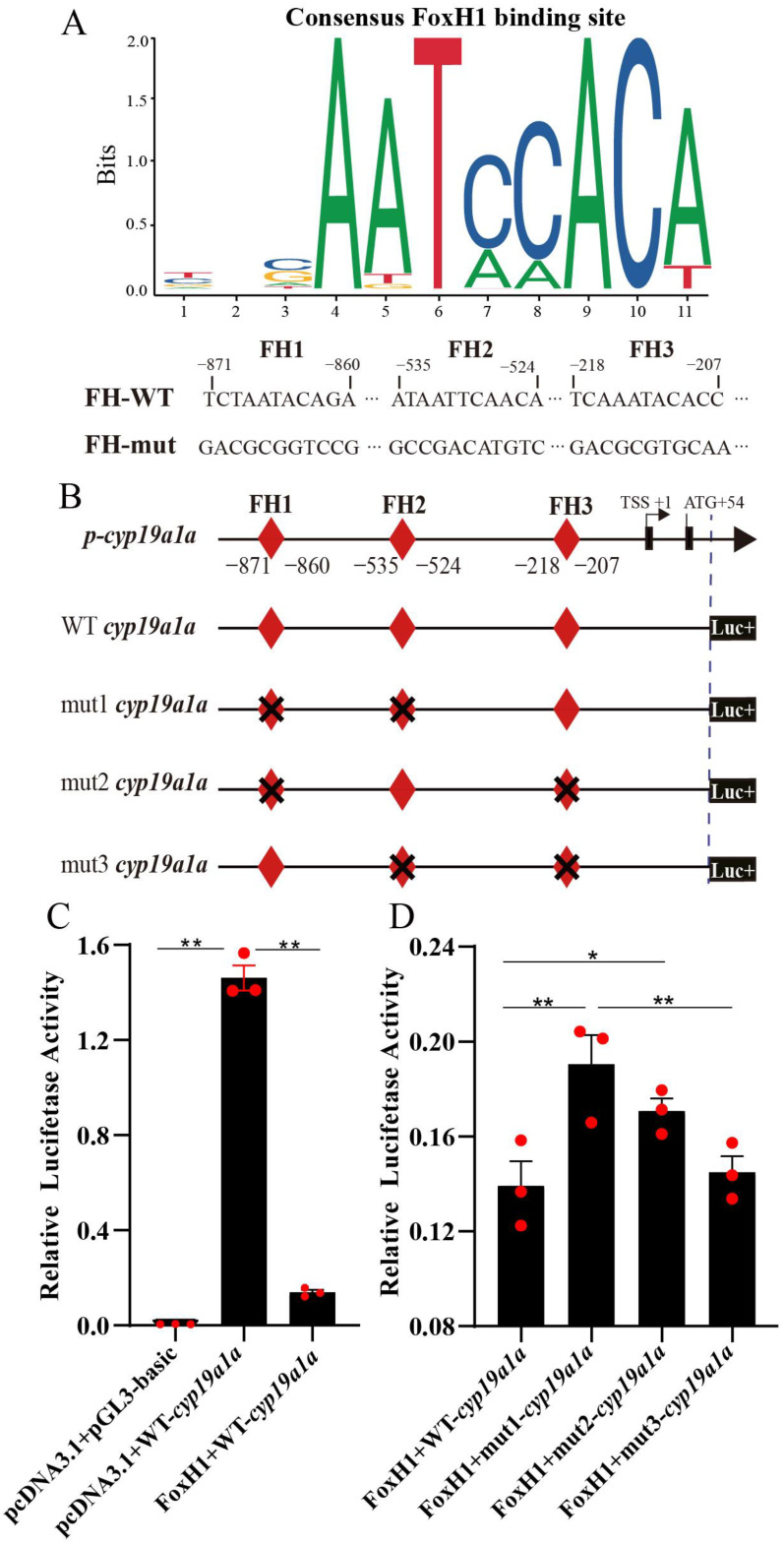
FoxH1 acts as a transcription factor and represses *cyp19a1a* promoter transcriptional activity. (**A**) Marker sequence of the FoxH1 binding site based on the JASPAR database and FoxH1 binding site sequence on the promoter of *cyp19a1a*. Mut indicates an FH mutation. (**B**) Schematic showing that different loci of the *cyp19a1a* promoter were cloned and inserted into the pGL3 vector. Potential FH sites are indicated by red diamonds, and the transcription start site (TSS) was counted as +1. Cross indicates the FH site is mutated. (**C**), The activity of *cyp19a1a* luciferase reporters in CHO cells with or without FoxH1 overexpression was measured. (**D**) The activity of wild-type and mutant-type *cyp19a1a* luciferase reporters in CHO cells overexpressing FoxH1 was measured. The results are presented as the means ± SEMs (*n* = 3). * *p* < 0.05; ** p < 0.01.

As shown in Figure 7A,B, three FoxH1 binding sites were identified in the *cyp19a1a* promoter by JASPAR online software (2022, the 9th release of the open-access database of transcription factor binding profiles), namely, FH1 (−871/−860), FH2 (−535/−524), and FH3 (−218/−207). To investigate whether FoxH1 was a transcription factor of the *cyp19a1a* gene, wild-type and mutated luciferase reporter vectors of *cyp19a1a* were constructed and cotransfected with pcDNA3.1-FoxH1 into CHO cells. Luciferase activity analysis revealed that FoxH1 overexpression significantly decreased the promoter activity of the *cyp19a1a* gene (*p* < 0.01) (Figure 7C). Moreover, the luciferase activity of the *cyp19a1a*-mut1 (*p* < 0.01) and *cyp19a1a*-mut2 (*p* < 0.05) vectors were significantly increased in FoxH1-overexpressing cells compared to control cells, whereas the promoter activity of *cyp19a1a*-mut3 showed no change (Figure 7D), suggesting that FoxH1 regulated the transcriptional activity of *cyp19a1a* through the FH1 and FH2 motifs.

## 3. Discussion

FoxH1 family members have been described in many vertebrate groups, including mammals [27], amphibians [18], and teleosts [28]. These proteins showed high homology in the Forkhead DNA binding domain and SID but very little conservation outside those domains. In the present study, we cloned 1533 bp cDNA sequences and characterized them. The results showed that *M. albus* FoxH1 is highly conserved between bony fish and amphibians, including species such as *A. latus*, *Oryzias latipes*, and *Xenopus laevis,* especially in the Forkhead domain and FoxH1 domain (FM1/2). These results imply that FoxH1 may have a conserved function in bony fish.

Previous studies have demonstrated that *foxh1* is a maternal and zygotic gene that plays an important role in early embryonic development. Similar to the results of studies on zebrafish [28], Xenopus [18,29], and mice, *M. albus foxh1* is expressed maternally. Gonad RNA-seq data of nile tilapia [12], rock bream [22], and yellowfin seabream [23] have shown that *foxh1* expression levels are significantly higher in the ovary than in the testis. Notably, yellowfin seabream is a protandrous hermaphroditic fish, and *foxh1* expression levels in the ovary and ovo-testis are approximately 20 times higher than those in the testis [23]. As determined in the present study, *M. albus foxh1* is a sexually dimorphic gene, and its expression levels in the ovary are three times higher than those in the testis. Moreover, *foxh1* expression levels increase from PG to EV and decrease from EV to OM during ovarian development. This finding was consistent with the TGF-β family *bmpr2* [30], *smad2* [24] and *smad3* (unpublished data from our lab) expression patterns in *M. albus* ovaries. These results reveal that *foxh1* may be involved in early folliculogenesis and previtellogenesis as a Smad2/3 transcriptional partner mediating TGF-β signaling.

Gonadotropins participate in ovarian development by regulating numerous gene networks, such as those related to steroid synthesis, cell proliferation, and differentiation [31]. The expression levels of FoxH1 remained stable after pregnant mare serum gonadotropin (PMSG) and hCG treatment, and FoxH1 was localized in the newly formed corpus luteum, but its expression decreased as the corpus luteum degenerated [21]. In the present study, neither time nor dose dependency was observed in either FSH or hCG treatments. In the FSH group, FSH stimulated *foxh1* expression, but the stimulation weakened with time. However, the effect of hCG on *foxh1* expression was not regular. FSH in teleosts is primarily involved in the control of oocyte growth [2,32]. Fshb immunoreactive signals and *fshb* mRNA in the *M. albus* pituitary increased at the onset of secondary oocyte growth, indicating that FSH is key to the onset of first puberty and vitellogenesis [33]. This is consistent with *foxh1* expression patterns during ovarian development. Taken together, these results suggest that *foxh1* is an FSH-responsive gene and might play important roles in previtellogenesis, follicular cell layer formation, and FSH-modulated ovary development.

During zebrafish oogenesis, *foxh1* mRNA changed its localization from the vegetal Balbiani body to the animal pole between stage I and II oocytes [34]. FoxH1 was strongly expressed in oocytes and TCs throughout folliculogenesis in mouse ovaries [21]. In XX tilapia, *foxh1* signals were present in the cytoplasm of stage I and II oocytes in the ovary by in situ hybridization [12]. Moreover, *foxh1*^−/−^ XX tilapia oocytes failed to transition from phase II to phase III, and follicle cells were blocked from transitioning from one to two layers [35]. In the present study, FoxH1 was localized mainly in oocytes and GCs. Notably, the transfer of FoxH1 signals from oocytes to GCs followed subsequent development. FoxH1 was not observed in the cytoplasm of oocytes during vitellogenesis, whereas it was observed in GCs and TCs. Additionally, FoxH1 and Cyp19a1a colocalized in GCs. Considering these results together, the cell-specific expression pattern of FoxH1 in *M. albus* raises the possibility that FoxH1 may promote oocyte growth, GC proliferation, and steroid hormone synthesis.

FoxH1 acts as an activator or repressor, alone or in concert with other transcription factors, to regulate target genes. For example, FoxH1 activated *lim* gene transcription alone or in conjunction with Smad2/4 [36]. FoxH1 also interacted with Smad2/3 to activate downstream genes of Nodal signaling [37,38]. Furthermore, FoxH1 bound to a corepressor to repress *xrn1* gene transcription [39,40]. FoxH1 repressed androgen receptor (AR) transcriptional activity and colocalized with AR [41]. FoxH1 repressed the ligand-dependent and ligand-independent transcriptional activity of the estrogen receptor through the estrogen response element [42]. In the present study, the levels of *foxh1* in ovaries peaked at the EV stage. However, *cyp19a1a* mRNA levels were highest in the MLV stage [43]. FoxH1 and Cyp19a1a colocalized in GCs. In addition, three FoxH1-binding sites were predicted in the *cyp19a1a* promoter, namely, FH1 (−871/−860), FH2 (−535/−524), and FH3 (−218/−207). pcDNA3.1-FoxH1 and *cyp19a1a*-Luc were cotransfected into CHO cells, and FoxH1 significantly suppressed transcription of the *cyp19a1a* gene. However, *cyp19a1a* transcriptional activity was significantly increased when FH1/2 and FH1/3 were mutated. In addition, the promoter activity of *cyp19a1a* mut-1 (FH1/2 mutant type) was higher than that of *cyp19a1a* mut-2 (FH1/3 mutant type). The transcriptional activity of *cyp19a1a* mut-3 (FH2/3 mutant type) did not change. These results suggest that FoxH1 may act as a repressor to regulate the promoter activity of *cyp19a1a* via FH1 and FH2. In contrast, Cyp19a1a expression levels were significantly reduced in *foxh1*^−/−^ XX tilapia, possibly because the transition from stage II to III and follicular cells from one to two layers were blocked [4]. It is well known that teleost aromatase is synthesized predominantly at the follicular cell layer [5]. In addition, ovarian transcriptomics of *foxh1*^−/−^ XX tilapia have shown decreased *foxl2* expression and increased *dmrt1* expression [4]. FoxL2 was a transcriptional activator of *cyp19a1a* in tilapia [6] and *M. albus* [7]. Dmrt1 inhibited the transcription of *cyp19a1a* in tilapia [8]. Therefore, the decrease in *cyp19a1a* in *foxh1*^−/−^ tilapia may be caused by the FoxH1 regulated gene network. In addition, no study has reported the regulatory relationship between FoxH1 and *cyp19a1a*. However, the present study determined that there is a regulatory relationship between them, and in vivo studies are needed to reveal the regulatory mechanisms and the effects on the downstream pathways and gonadal development, which will be the focus of future studies.

To date, most studies on FoxH1 functions have focused on the role of FoxH1 during embryogenesis, while little is known about the roles of FoxH1 reproduction. To date, most studies on FoxH1 functions have focused on the role of FoxH1 during embryogenesis, while little is known about the roles of FoxH1 reproduction. The ricefield eel is a protogynous hermaphrodite fish for which it is difficult to phenotypically differentiate sex in the non-spawning season; sex discrimination requires histological observation or detection of the expression of sex-specific genes, such as *cyp19a1a* (a female-specific gene) and *dmrt1* (a male-specific gene). We found evidence that FoxH1 is a sexually dimorphic gene that is highly expressed in the ovaries and expressed at low levels in the testes; thus, *foxh1* can be used as a female marker gene. Furthermore, FoxH1 acts as a suppressor of *cyp19a1a* transcription and maintains its expression in sexually mature females, potentially prolonging the spawning cycle of females. These findings provide new insights for artificial sex control and for the improvement of spawning quality. Overall, this study demonstrates that FoxH1 regulates *cyp19a1a* transcription in vitro, but further in vivo studies are needed to understand the role of FoxH1 in bony fish ovarian development.

## 4. Materials and Methods

### 4.1. Sample Collection and Preparation

The wild *M. albus* (*n* = 100, body length = 34.93 ± 4.52 cm and body weight = 37.99 ± 21.88 g) used in the present study were obtained from a local market in Chengdu, Sichuan. These fish were kept under the natural temperature and photoperiod in the laboratory. All experimental procedures involving animal research were subject to approval and performed in accordance with the guidelines of the ethics committee (Approval No. 20190031).

Fish were anesthetized with 0.02% tricaine buffer (80 μg/L) (Sigma, West Hollywood, LA, USA) for 10 min after a 24 h fast, and the tissues, including half of the gonads, pituitary, eyes, heart, kidneys, intestines, spleen, muscles, and blood, were collected and immediately stored in liquid nitrogen. A portion of the fresh gonads was immediately fixed in Bouin’s solution for 24 h and embedded in paraffin. Sections were serially cut at a thickness of 5 μm using a slicer (Leica, Nussloch, Germany) and stained with hematoxylin/eosin. The histological classification of the gonad, including the PG, previtellogenic stage (PV), EV, MLV, and OM, has been described previously [44].

### 4.2. RNA Extraction and cDNA Synthesis

Total RNA was extracted using TRIzol (Invitrogen, Chicago, IL, USA) following the manufacturer’s instructions. A RevertAid First-strand cDNA Synthesis Kit (Thermo Scientific, Waltham, MA, USA) was used to generate cDNA according to the manufacturer’s instructions. cDNA quality was verified by the successful amplification of *ef1α* and *rpl17* [26].

### 4.3. Cloning of M. albus foxh1 cDNA

Specific primers (*foxh1*-F1, *foxh1*-R1, *foxh1*-F2, and *foxh1*- R2 (Table 1)) were designed to clone *foxh1* based on the coding sequence from the *M. albus* genome (Accession No: 109965524). After an initial 0.5 min denaturation at 95 °C, PCR was conducted for all of the above genes with the following cycling conditions: 35 cycles of 94 °C for 0.5 min, 55 °C for 0.5 min, and 72 °C for 0.5 min, with a final extension at 72 °C for 30 min. All target products were ligated into pMD19-T (TaKaRa, Dalian, China) and sequenced by TsingKE Biological Technology Company Limited (Chengdu, Sichuan, China).

Multiple alignments of the amino acid sequences were conducted with ClustalX 1.83. Based on the deduced amino acid sequences, a phylogenetic tree was constructed via the neighbor-joining method with bootstrap values calculated from 1000 replicates in the MEGA 11 software package.

### 4.4. Quantitative Real-Time Polymerase Chain Reaction (qRT–PCR)

cDNA was obtained from gonads at the five developmental stages and from other tissues. qRT–PCR was performed with a Bio-Rad CFX Connect system (Bio-Rad, Chicago, IL, USA) to determine the expression levels of *foxh1*. The sequences of the primers are listed in Table 1. The cycling parameters were as follows: 95 °C for 5 min followed by 40 amplification cycles of 95 °C for 10 s, 59 °C for 15 s, and 72 °C for 20 s. To minimize variation due to differences in cDNA loading, the geometric mean expression levels of *rpl17* and *ef1α* were utilized to normalize the expression levels of the target genes. Target gene expression was calculated with the equation C*_target gene_*/$\sqrt{C_{\mathrm{ef}1a}\times C_{\mathrm{rpl}17}}$.

### 4.5. Immunofluorescence

Immunofluorescence was used to locate FoxH1 and Cyp19 in paraffin-embedded ovarian tissues at the EV and MLV stages. Briefly, ovarian sections were deparaffinized, rehydrated, and subjected to high-temperature (95–98 °C) antigen retrieval for 10 min with EDTA (pH 8.0). Then, the sections were blocked in 3% BSA for 30 min at room temperature and incubated with primary antibodies overnight at 4 °C. The primary antibodies included FoxH1 (Genetex, GTX17182, 1:1000) and Cyp19a1a (Genetex, GTX18995, 1:1000). Subsequently, the sections were incubated with fluorophore-conjugated goat anti-rabbit secondary antibodies (Servicebio, GB23303, 1:2000) for 2 h at 37 °C. Finally, the sections were coverslipped using anti-fade fluorescent mounting medium (Servicebio, G1221-5ML), imaged using Pannoramic 250 fully automated digital scanning microscope(3DHISTECH), and observed with the CaseViewer application(3DHISTECH).

### 4.6. Expression Patterns of foxh1 after Incubation of Ovaries with hCG and FSH In Vitro

Gonads of female *M. albus* at the MLV stage were washed and dissected in Leibovitz L-15 medium (Gibco, Carlsbad, CA, USA) on ice. Ovarian tissues (50–100 mg) were placed in 24-well tissue culture dishes in 1 mL of Leibovitz L-15 medium (0.1 U/mL penicillin and 0.1 mg/mL streptomycin) with FSH (0.05, 1.0, and 5.0 ng/mL), hCG (10, 50, and 100 IU/mL), or saline solution (control group), and then incubated at 28 °C for 1, 2, 4, and 10 h.

### 4.7. Sequence Analysis

The promoter sequence (1866 bp) of *cyp19a1a* was obtained from Prof. Zhang, Institute of Aquatic Economic Animals, Sun Yat-Sen University, China [45,46]. The JASPAR (https://jaspar.genereg.net/) (accessed on 1 January 2023) and PROMO (http://alggen.lsi.μpc.es/cgi-bin/promo_v3/promo/promoinit) (accessed on 1 January 2023) online software programs were employed to predict possible FoxH1-binding sites in the *cyp19a1a* promoter.

### 4.8. Plasmid Construction and Dual-Luciferase Reporter Assays

To generate luciferase reporters, fragments of the *cyp19a1a* promoter (1866 bp) were amplified, cloned, and inserted into a pGL3-Basic reporter vector between the *NheI* and *XhoI* restriction sites. For FoxH1 expression vector construction, the *foxh1* full-length coding sequence (1533 bp) of *M. albus* was amplified, double-digested with *NheI* and *EcoRI*, and then cloned and inserted into the pcDNA3.1 vector. To further evaluate the effects of FoxH1 on the transcriptional activity of *M. albus cyp19a1a,* its promoter containing wild-type FoxH1 binding sites (FHs) was amplified, cloned, and inserted into the pGL3-Basic reporter vector between the *KpnI* and *XhoI* restriction sites. Additionally, FH mutant-type vectors were constructed by using a TaKaRa MutanBEST Kit (#R401, TaKaRa, Beijing, China) according to the manufacturer’s instructions with the wild-type plasmids as templates. The sequences of wild-type and mutant FH binding sites are shown in Table 2. All recombinant plasmids were constructed by Bioengineering (Shanghai, China) Co., Ltd. and verified by Sanger sequencing. The primers for plasmid construction are listed in Table 1.

For luciferase activity detection, after transfection for 48 h, the cells were harvested, and their lysates were collected for dual-luciferase analysis with a Dual-Luciferase Reporter Assay System (#E1910, Promega, Madison, WI, USA) following the kit’s manual. The GloMax detection system (Promega) was used to measure firefly and Renilla luciferase activity in cell lysates.

### 4.9. Statistical Analysis

Statistical analyses were performed by using GraphPad Prism v8.0 software (GraphPad, California, CA, USA) and SPSS v20.0 (IBM, Armonk, NY, USA). All data are presented as the mean ± SEM from three independent experiments. Comparisons among three or more different groups were conducted by using one-way ANOVA followed by Duncan’s multiple comparisons test. * p < 0.05 was considered to indicate statistical significance, and the significance levels are stated in the corresponding figure legends.

## 5. Conclusions

In summary, foxh1 is a sexually dimorphic gene. The *foxh1* levels in the ovaries were three times those in the testes, and they were regulated by the gonadotropins FSH and hCG in vitro. FoxH1 colocalized with Cyp19a1a in the oocytes and GCs of middle and late vitellogenic follicles. Furthermore, three FoxH1 binding sites were identified in the proximal promoter of *cyp19a1a*, and FoxH1 overexpression significantly attenuated the activity of the *cyp19a1a* promoter in CHO cells. This study demonstrates that FoxH1 regulates *cyp19a1a* transcription in vitro, but further in vivo studies are needed to understand the role of FoxH1 in bony fish ovarian development.

## Figures and Tables

**Table 1:** Primers used for cloning and mRNA expression analysis.

Gene Name	Primer	Sequence (5′-3′)	Product Size (bp)
*foxh1*	F1	CGATTACGCAGCGGGATT	1167
	R1	GAGGCACTATGAGCAGAGGATG	
	F2	CTGAGCTACCCTCTGACCCT	985
	R2	CACTGTCTGTGGATCGGCAT	
	*qF*	CCCACCACAGGAGGACTT	198
	*qR*	GCAGAGGCACTATGAGCAG	
*ef1α*	F	CGCTGCTGTTTCCTTCGTCC	102
	R	TTGCGTTCAATCTTCCATCCC	
*rpl17*	F	GTTGTAGCGACGGAAAGGGAC	160
	R	GACTAAATCATGCAAGTCGAGGG	
p*cyp19a1a*	F	GCTCTTACGCGTGCTAGCCACCACTGACTTTGGTACAGAAG	For pGL3-basic construction
	R	TAGATCGCAGATCTCGAGGTTCACAAGCAGGGATCAGAT	

F: forward primers; R: reverse primers.

**Table 2:** Mutation methods for FoxH1 mutant plasmids.

Plasmids	Mutation Site (5′-3′)	Post-Mutation Site
FoxH1-mut1	TCTAATACAGA	gacgcggtccg
FoxH1-mut2	ATAATTCAACA	gccgacatgtc
FoxH1-mut3	TCAAATACACC	gacgcgtgcaa

## Data Availability

The datasets supporting the conclusions of this article are included within the article.

## Supplementary Materials

The supporting information can be downloaded at: https://www.mdpi.com/article/10.3390/ijms241813712/s1.

## References

[1] Rajakumar A., Senthilkumaran B. (2020). Steroidogenesis and its regulation in teleost—A review. Fish. Physiol. Biochem..

[2] Lubzens E., Young G. (2010). Oogenesis in teleosts: How eggs are formed. Gen. Comp. Endocrinol..

[3] Hannenhalli S., Kaestner K.H. (2009). The evolution of Fox genes and their role in development and disease. Nat. Rev. Genet..

[4] Dai S., Qu L. (2021). Toward a mechanistic understanding of DNA binding by forkhead transcription factors and its perturbation by pathogenic mutations. Nucleic Acids Res..

[5] Wang D.S., Kobayashi T. (2007). Foxl2 up-regulates aromatase gene transcription in a female-specific manner by binding to the promoter as well as interacting with ad4 binding protein/steroidogenic factor 1. Mol. Endocrinol..

[6] Ji X., Bu S. (2022). Identification of SF-1 and FOXL2 and Their Effect on Activating P450 Aromatase Transcription via Specific Binding to the Promoter Motifs in Sex Reversing *Cheilinus undulatus*. Front. Endocrinol..

[7] Si Y., Ding Y. (2016). DNA methylation level of cyp19a1a and Foxl2 gene related to their expression patterns and reproduction traits during ovary development stages of Japanese flounder (*Paralichthys olivaceus*). Gene.

[8] Ning Y., Fan M. (2021). Two Foxo1 homologues in the orange-spotted grouper *Epinephelus coioides*: Sequences, expression, and possible involvement in the activation of cyp19a1a expression in the ovary. Fish. Physiol. Biochem..

[9] Liu Q., Zhang Y. (2016). Foxo3b but not Foxo3a activates cyp19a1a in *Epinephelus coioides*. J. Mol. Endocrinol..

[10] Zhang W., Lu H. (2012). Isolation and characterization of cyp19a1a and cyp19a1b promoters in the protogynous hermaphrodite orange-spotted grouper (*Epinephelus coioides*). Gen. Comp. Endocrinol..

[11] Huang W., Zhou L. (2009). Expression pattern, cellular localization and promoter activity analys is of ovarian aromatase (Cyp19a1a) in protogynous hermaphrodite red-spotted grouper. Mol. Cell. Endocrinol..

[12] Yuan J., Tao W. (2014). Genome-wide identification, phylogeny, and gonadal expression of fox genes in *Nile tilapia*, *Oreochromis niloticus*. Fish. Physiol. Biochem..

[13] Chen X., Rubock M.J. (1996). A transcriptional partner for MAD proteins in TGF-beta signalling. Nature.

[14] Chen X., Weisberg E. (1997). Smad4 and FAST-1 in the assembly of activin-responsive factor. Nature.

[15] Charney R.M., Forouzmand E. (2017). Foxh1 Occupies cis-Regulatory Modules Prior to Dynamic Transcription Factor Interactions Controlling the Mesendoderm Gene Program. Dev. Cell.

[16] Slagle C.E., Aoki T. (2011). Nodal-dependent mesendoderm specification requires the combinatorial activities of FoxH1 and Eomesodermin. PLoS Genet..

[17] Attisano L., Silvestri C. (2001). The transcriptional role of Smads and FAST (FoxH1) in TGFbeta and activin signalling. Mol. Cell Endocrinol..

[18] Howell M., Inman G.J. (2002). A novel Xenopus Smad-interacting forkhead transcription factor (XFast-3) cooperates with XFast-1 in regulating gastrulation movements. Development.

[19] Pogoda H.M., Solnica-Krezel L. (2000). The zebrafish forkhead transcription factor FoxH1/Fast1 is a modulator of nodal signaling required for organizer formation. Curr. Biol..

[20] Pei W., Noushmehr H. (2007). An early requirement for maternal FoxH1 during zebrafish gastrulation. Dev. Biol..

[21] Wang G., Liu L. (2016). Expression and distribution of forkhead activin signal transducer 2 (FAST2) during follicle development in mouse ovaries and pre-implantation embryos. Acta Histochem..

[22] Li H., Zhu Q. (2021). Identification and Characterization of Dimorphic Expression of Sex-Related Genes in Rock Bream, a Fish With Multiple Sex Chromosomes. Front. Genet..

[23] Li S., Lin G. (2020). Gonadal Transcriptome Analysis of Sex-Related Genes in the Protandrous Yellowfin Seabream (*Acanthopagrus latus*). Front. Genet..

[24] He Z., Deng F. (2020). Expression and regulation of Smad2 by gonadotropins in the protogynous hermaphroditic ricefield eel (*Monopterus albus*). Fish. Physiol. Biochem..

[25] He Z., Deng F. (2021). Molecular characterization, expression, and H_2_O_2_ induction of p53 and mdm2 in the ricefield eel, *Monopterus albus*. Aquac. Rep..

[26] He Z., Deng F. (2022). Crosstalk between sex-related genes and apoptosis signaling reveals molecular insights into sex change in a protogynous hermaphroditic teleost fish, ricefield eel *Monopterus albus*. Aquaculture.

[27] Zhou S., Zawel L. (1998). Characterization of human FAST-1, a TGF beta and activin signal transducer. Mol. Cell.

[28] Boggetti B., Argenton F., Haffter P., Bianchi M.E., Cotelli F., Beltrame M. (2000). Cloning and expression pattern of a zebrafish homolog of forkhead activin signal transducer (FAST), a transcription factor mediating Nodal-related signals. Mech. Dev..

[29] Kofron M., Puck H., Standley H., Wylie C., Old R., Whitman M., Heasman J. (2004). New roles for FoxH1 in patterning the early embryo. Development.

[30] He Z., Zheng L. (2022). Expression Patterns and Gonadotropin Regulation of the TGF-β II Receptor (Bmpr2) during Ovarian Development in the Ricefield Eel *Monopterus albus*. Int. J. Mol. Sci..

[31] Rimon-Dahari N., Yerushalmi-Heinemann L. (2016). Ovarian Folliculogenesis. Results Probl. Cell Differ..

[32] Li J., Cheng C.H.K. (2018). Evolution of gonadotropin signaling on gonad development: Insights from gene knockout studies in zebrafish. Biol. Reprod..

[33] Wu Y., He Z. (2012). Ontogeny of immunoreactive Lh and Fsh cells in relation to early ovarian differentiation and development in protogynous hermaphroditic ricefield eel *Monopterus albus*. Biol. Reprod..

[34] Bontems F., Stein A. (2009). Bucky ball organizes germ plasm assembly in zebrafish. Curr. Biol..

[35] Tao W., Shi H. (2020). Homozygous mutation of foxh1 arrests oogenesis causing infertility in female *Nile tilapia*. Biol. Reprod..

[36] Watanabe M., Rebbert M.L. (2002). Regulation of the Lim-1 gene is mediated through conserved FAST-1/FoxH1 sites in the first intron. Dev. Dyn..

[37] Silvestri C., Narimatsu M. (2008). Genome-wide identification of Smad/Foxh1 targets reveals a role for Foxh1 in retinoic acid regulation and forebrain development. Dev. Cell.

[38] Chiu W.T., Charney Le R. (2014). Genome-wide view of TGFβ/Foxh1 regulation of the early mesendoderm program. Development.

[39] Reid C.D., Steiner A.B. (2016). FoxH1 mediates a Grg4 and Smad2 dependent transcriptional switch in Nodal signaling during *Xenopus mesoderm* development. Dev. Biol..

[40] Halstead A.M., Wright C.V. (2015). Disrupting Foxh1-Groucho interaction reveals robustness of nodal-based embryonic patterning. Mech. Dev..

[41] Chen G., Nomura M. (2005). Modulation of androgen receptor transactivation by FoxH1. A newly identified androgen receptor corepressor. J. Biol. Chem..

[42] Yum J., Jeong H.M. (2009). PKA-mediated stabilization of FoxH1 negatively regulates ERalpha activity. Mol. Cells.

[43] Zhang Y., Zhang W. (2008). Two cytochrome P450 aromatase genes in the hermaphrodite ricefield eel *Monopterus albus*: mRNA expression during ovarian development and sex change. J. Endocrinol..

[44] He Z., Ma Z. (2022). Circular RNA expression profiles and CircSnd1-miR-135b/c-foxl2 axis analysis in gonadal differentiation of protogynous hermaphroditic ricefield eel *Monopterus albus*. BMC Genom..

[45] Yan T., Lu H. (2021). Nr5a homologues in the ricefield eel Monopterus albus: Alternative splicing, tissue-specific expression, and differential roles on the activation of cyp19a1a promoter in vitro. Gen. Comp. Endocrinol..

[46] Zhang Y., Zhang S. (2013). Epigenetic modifications during sex change repress gonadotropin stimulation of cyp19a1a in a teleost ricefield eel (*Monopterus albus*). Endocrinology.

