# Supplementary material for: FoxH1 Represses the Promoter Activity of cyp19a1a in the Ricefield Eel (Monopterus albus)
Source: Int J Mol Sci. 2023 Sep 5;24(18):13712. doi: 10.3390/ijms241813712 (PMC10531137; doi:10.3390/ijms241813712)
Supplement: Supplementary file 1 [file ijms-24-13712-s001.zip › ijms-2507361-supplementary.pdf]

Supplementary figures

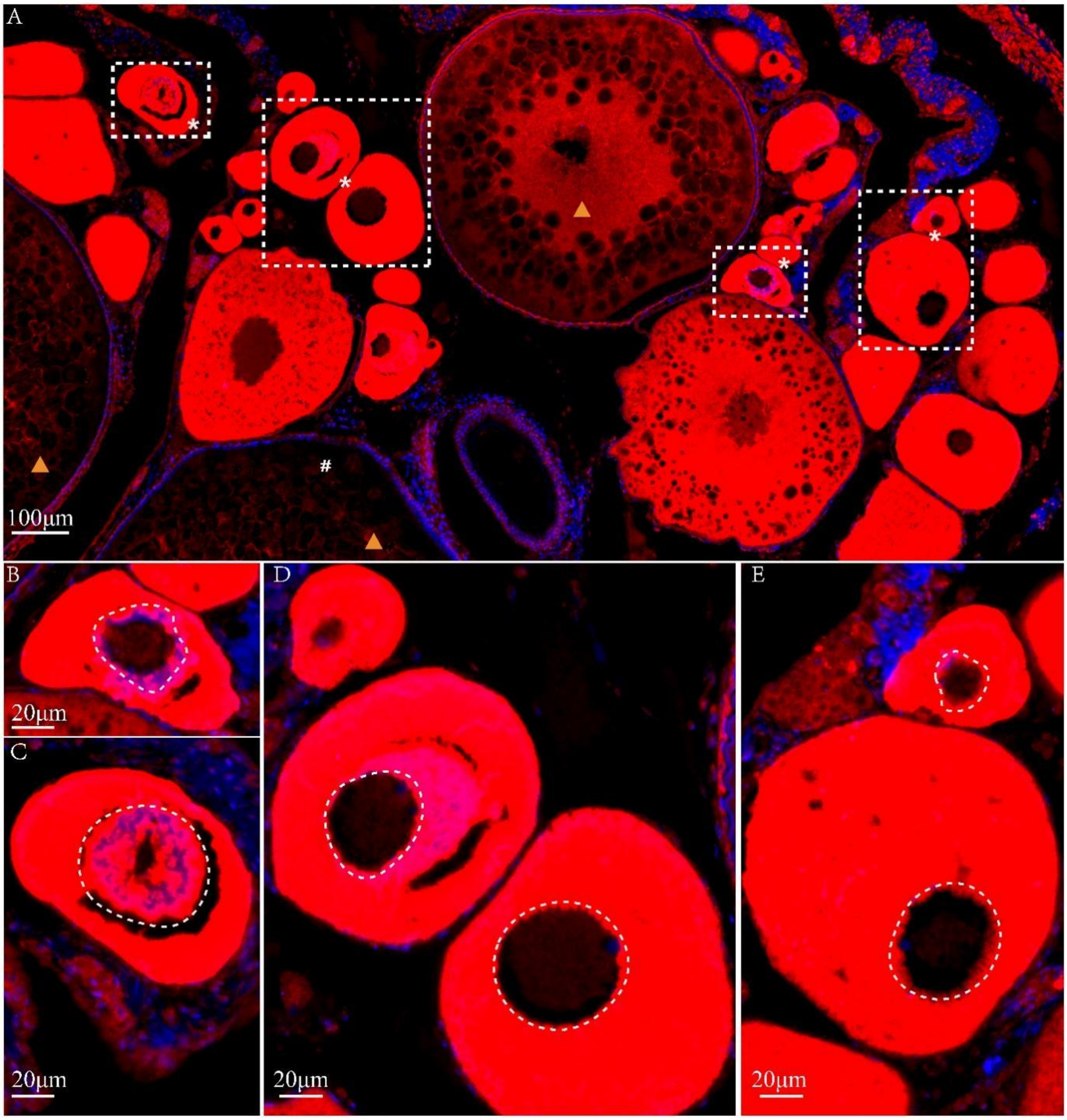

Figure S1. (**A**) Colocalization of immunofluorescent FoxH1 in EV *Monopterus albus ovaries*. (**B–E**) are the dotted boxes in Figure S1A. Nuclei are labeled with DAPI (blue). Asterisk (*), primary growth oocytes (PGOs); pound (#), cortical alveoli stage oocytes (CAOs); triangle (△), early vitellogenic-stage oocytes (EVOs).

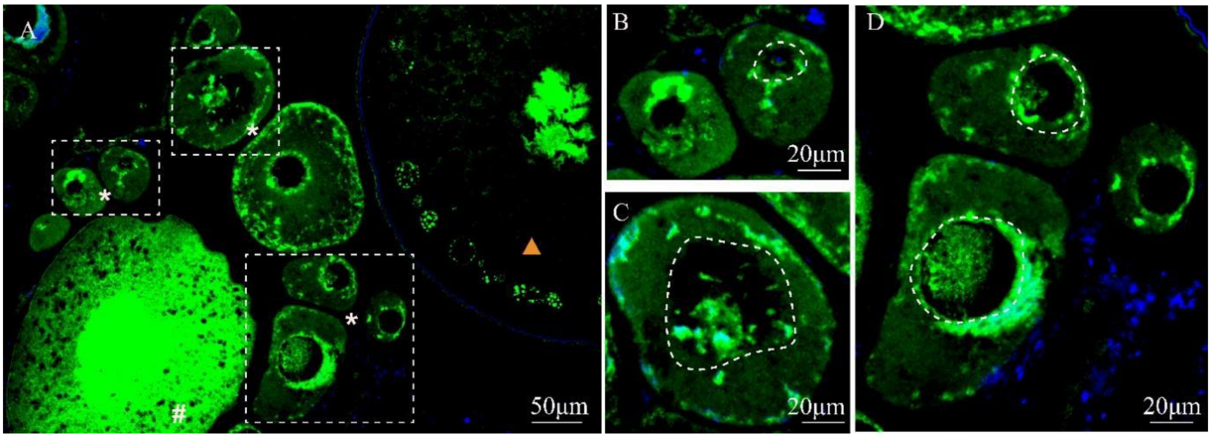

Figure S2. (**A**) Colocalization of immunofluorescent Cyp19a1a in EV *Monopterus albus ovaries*. (**B–D**) are the dotted boxes in Figure S2A. Nuclei are labeled with DAPI (blue). Asterisk (*), primary growth oocytes (PGOs); pound (#), cortical alveoli stage oocytes (CAOs); triangle (△), early vitellogenic-stage oocytes (EVOs).
